# Efficacy of Oral Etoposide in Pretreated Metastatic Breast Cancer: A Multicenter Phase 2 Study: Erratum

**DOI:** 10.1097/01.md.0000467314.84255.6b

**Published:** 2015-06-26

In the article “Efficacy of Oral Etoposide in Pretreated Metastatic Breast Cancer: A Multicenter Phase 2 Study”,^1^ which appeared in Volume 94, Issue 17 of *Medicine*, the legend for Figure 2 was presented incorrectly. The green line should represent Internal organ metastasis, and the blue line should represent No internal organ metastasis. The article has been corrected online.
